# Editorial: miRNAs and inflammation: from biogenesis to therapeutic option

**DOI:** 10.3389/fimmu.2023.1296589

**Published:** 2023-10-03

**Authors:** Alireza Tahamtan, Saeed Samadizadeh, Vahid Salimi, Lucia Natarelli, Britt Nakstad

**Affiliations:** ^1^ Department of Microbiology, Golestan University of Medical Sciences, Gorgan, Iran; ^2^ Infectious Diseases Research Center, Golestan University of Medical Sciences, Gorgan, Iran; ^3^ Department of Virology, Tehran University of Medical Sciences, Tehran, Iran; ^4^ Institute for Cardiovascular Prevention, Ludwig-Maximillians University, Munich, Germany; ^5^ Department of Paediatrics and Adolescent Health, University of Botswana, Gaborone, Botswana; ^6^ Division of Paediatrics and Adeolescent Medicine, Institute of Clinical Medicine, University of Oslo, Oslo, Norway

**Keywords:** miRNA, inflammatory disease, inflammation, noncoding RNA, gene expression

Research on miRNAs, a class of short non-coding RNA molecules composed of ~18-25 nucleotides, dates back to the early 1990s, with discoveries representing their post-transcriptional regulatory effects (1). The new trending area has since then improved by further studies unraveling the functional roles of miRNAs in diverse biological mechanisms, such as development, differentiation, apoptosis, and disease (2). miRNAs also have a crucial role in regulating inflammation by controlling the expression of genes related to immune responses. They can either promote or inhibit inflammation depending on the specific miRNA and its target genes (2). Investigating the involvement of miRNAs in inflammatory conditions provides valuable insights into their various biogenesis mechanisms and therapeutic options for a wide range of diseases. This Research Topic focuses on the latest advances and discoveries in miRNAs and inflammation, essential for deciphering the intricacies of their biogenesis and therapeutic potential.

miRNA biogenesis is vital to study as a prerequisite for understanding post-transcriptional gene fine-tuning, its role in health and disease, and therapeutic strategies. The classical miRNA biogenesis pathway depends on the enzymes Drosha and Dicer, producing mature miRNAs from pri-miRNA and pre-miRNA transcripts (3). In this concept, pre-miRNAs are released into the cytoplasm by Exportin 5 for cytoplasmic processing and mature miRNA formation. Similarly, in alternative biogenesis pathways, including the Mirtron pathway or those involving non-canonical processing, miRNA maturation transpires in the cytoplasm (4), raising the initial speculation of scientists that mature miRNAs are restricted to the cytoplasm. Nevertheless, emerging evidence has shown the presence of specific mature miRNAs in the nucleus, suggesting the hypothesis that some mature miRNAs can re-enter the nucleus. On this Research Topic, Hu et al. have reviewed studies on nuclear miRNAs, describing their effects on their counterparts’ biogenesis, gene transcription, and associated signal transduction. Such subcellular localization of miRNAs may refer to their compartmentalized nature, posing functional implications in the nucleus and cytoplasm. Accordingly, it is demonstrated that nuclear miRNAs modulate gene expression by targeting elements within gene promoter or enhancer regions. These processes include miRNAs targeting TATA-box in promoters and interactions with enhancers, mediated by RNA-nuclear activating miRNAs (NamiRNAs) during target gene transcriptional regulation (5, 6). Moreover, it is shown that nuclear miRNA regulation is associated with signaling pathways related to general metabolic processes, lysosomal biogenesis, and autophagy, implying potential arenas for therapeutic applications.

The study by Hu et al. also provides insights into the cross-talk and modulatory networks between nuclear miRNAs. New evidence suggests that miRNAs regulate the expression and functions of both their own and other miRNAs through positive and negative feedback loops. For instance, mature Let-7 miRNA can create a positive feedback loop through a conserved complementary site on its own primary mRNA. miRNAs can similarly regulate the biogenesis of other miRNAs by binding to their primary mRNAs in the nucleus. In the study by Chithanathan et al., they discovered that miRNA-146a and miRNA-146b engage in cross-talk to regulate LPS-induced neuroinflammation. Using a miR-146b deficient mice model, they demonstrated that miR-146b deficiency results in the attenuation of LPS-induced neuroinflammation. Intriguingly, they also observed a relatively substantial increase in miR-146a levels in Mir146b-/- mice compared to the wild-type group following LPS exposure. The compensatory upregulation of miR-146a is found to be due to the over-expression of a miR-146a transcription inducer, interferon regulatory factor 7 (Irf7), in the absence of miR-146b.

Leveraging insights from studies in miRNA biogenesis can facilitate the development of precision therapies for inflammatory diseases, such as intestinal inflammation. Boros et al. reported a global alteration in the landscape of microRNAome in a rat model of inflammatory bowel disease (IBD). Performing a genome-wide microRNAome analysis, they identified a significant decrease in the expression levels of the miR-200 family members and miR-27b, correlating with reduced levels of their enhancers (HNF1B, E2F1), as well as elevated expression of their repressors (ZEB2, NFKB1) and their target genes (ZEB2, RUNX1). Boros et al. further signified the interplay between transcription factors and miRNAs in the IBD pathomechanism, as well as the role of the dysregulated miRNA expression at all levels of their biogenesis in the progress of IBD-related colorectal cancer. Peng et al. also confirmed miR-200a down-regulation in a mouse model of dextran sulfate sodium (DSS)-induced colitis. Their study indicated that miR-200a protected against DSS-induced colonic damage by activating the Keap1/Nrf2 signaling pathway, offering an innovative therapeutic approach to alleviate oxidative stress, apoptosis, and inflammatory responses in ulcerative colitis.

RNA-based therapeutics are also promising in diseases where macrophages have a significant role (2). For instance, allergic rhinitis (AR) is a chronic nasal disorder characterized by allergic Th2 inflammation, where M2 macrophages are known to contribute by stimulating Th2 responses (7). In this case, Wen et al. showed that MIR222HG attenuated macrophage M2 polarization and allergic inflammation in AR by targeting the miR146a-5p/TRAF6/NF-kB axis. MIR222HG is a miRNA-host-gene-derived long non-coding RNA acting as a competitive endogenous RNA (ceRNA) sponge, competing with miR146a-5p. These findings indicate a potential therapeutic role of MIR222HG through modulating macrophage polarization and pathogenesis in AR. In another study, Lin et al. highlighted the role of endothelial exosomes as a functional mediator in activating macrophages. Utilizing miRNA-seq and bioinformatics analysis, they illustrated that exosomes isolated from TNF-α-stimulated endothelial cells (exo-T) contained 104 differentially expressed miRNAs, with pathways being predominantly enriched in metabolic and MAPK/NF-κB signaling that are related to macrophage activation. Their study spotlights cell-to-cell communication mediated by exosomes through miRNAs that may exert modulatory effects on inflammatory diseases such as atherosclerosis.

To sum up, the articles published on this Research Topic navigated through the complexity of miRNA biogenesis and therapeutic potential in the context of inflammation. These findings have unveiled fresh discoveries on the roles and interactions of miRNAs, which can be translated into novel therapeutic strategies. Complementary to these investigations are studies aimed at developing efficient techniques for therapeutic applications.

## Author contributions

AT: Writing – original draft, Writing – review & editing. SS: Writing – original draft, Writing – review & editing. VS: Writing – original draft, Writing – review & editing. LN: Writing – original draft, Writing – review & editing. BN: Writing – original draft, Writing – review & editing.
